# Alder–Reilly Anomaly Leading to Incidental Diagnosis of MPS VII in an Asymptomatic Adult

**DOI:** 10.1002/jmd2.70128

**Published:** 2026-10-22

**Authors:** Margaretha G. M. Roemer, Monique Chevallier, Marie van Dijk, André B. P. van Kuilenburg, Kasper W. ter Horst, Janneke C. Zant

**Affiliations:** ^1^ Department of Clinical Chemistry Noordwest Ziekenhuisgroep Alkmaar the Netherlands; ^2^ Department of Clinical Chemistry Unilabs/Atalmedial Medical Diagnostic Centers Amsterdam the Netherlands; ^3^ Amsterdam UMC, Location AMC, Laboratory Genetic Metabolic Diseases Amsterdam the Netherlands; ^4^ Amsterdam UMC, Vrije Universiteit Amsterdam, Laboratory Genetic Metabolic Diseases Amsterdam the Netherlands; ^5^ Amsterdam Gastroenterology Endocrinology Metabolism (AGEM) Research Institute Amsterdam the Netherlands; ^6^ Department of Endocrinology and Metabolism Amsterdam UMC, Location AMC Amsterdam the Netherlands

## Abstract

Mucopolysaccharidosis type VII (MPS VII; Sly syndrome) is a rare autosomal recessive lysosomal storage disorder caused by deficiency of β‐glucuronidase due to pathogenic variants in the *GUSB* gene. Impaired degradation of glycosaminoglycans (GAGs) results in multiorgan dysfunction with highly variable clinical presentation, ranging from severe neonatal disease to attenuated adult phenotypes. We report an incidental diagnosis of MPS VII in a 31‐year‐old woman identified through routine laboratory testing. Review of a peripheral blood smear was prompted by automated hematology analyzer flags and revealed Alder–Reilly anomaly with characteristic aberrant leukocyte granulation. Subsequent biochemical analysis showed elevated urinary dermatan sulfate and heparan sulfate, alongside reduced β‐glucuronidase activity (~2%–3% of normal). Genetic testing of *GUSB* identified compound heterozygosity for a likely pathogenic frameshift variant (c.133_134del) and a missense variant (c.454G>A) previously associated with pseudodeficiency. Despite biochemical and genetic findings consistent with MPS VII, the patient exhibited no typical clinical manifestations, and detailed phenotyping showed no organ involvement. This case illustrates an atypical, asymptomatic presentation of MPS VII diagnosed in adulthood, in which Alder–Reilly anomaly served as a critical diagnostic clue. The combination of a null allele with a pseudodeficiency‐associated variant resulted in reduced enzyme activity without overt disease. These findings highlight the importance of peripheral blood smear evaluation in identifying lysosomal storage disorders and underscore the complexity of genotype–phenotype correlations in MPS VII.

## Key Points

- Alder–Reilly anomaly served as an incidental diagnostic clue to a lysosomal storage disorder in an otherwise clinically asymptomatic adult.
- Residual β‐glucuronidase activity of approximately 2%–3% appeared sufficient to prevent overt organ involvement yet insufficient to fully avert lysosomal storage within leukocytes.
- The combination of the p.Asp152Asn pseudodeficiency allele and a presumed null *GUSB* allele was associated with marked biochemical and morphological abnormalities without clinically apparent disease to date, highlighting the complexity of genotype–phenotype correlations in MPS VII.

## 1 Introduction

Mucopolysaccharidosis type VII (MPS VII; Sly syndrome) is a rare autosomal recessive disorder caused by a deficiency of the enzyme β‐glucuronidase due to biallelic pathogenic variants in the *GUSB* gene. The enzymatic defect impairs the degradation of glycosaminoglycans (GAGs), including chondroitin sulfate (CS), dermatan sulfate (DS), and heparan sulfate (HS), leading to accumulation of partially degraded GAGs within lysosomes of multiple tissues and organs. As a result, MPS VII is characterized by varying degrees of multisystem manifestations, including hepatosplenomegaly, cardiac valve disease, skeletal dysplasia (dysostosis multiplex), short stature, hydrops fetalis, and intellectual disability [1, 2].

Epidemiologically, MPS VII is one of the rarest MPS subtypes. The estimated birth prevalence ranges from ~1/345 000 to 1/5 000 000 with overall prevalence < 1 per 1 000 000, although the actual prevalence might be higher due to antenatal loss and early mortality [3].

Clinically, MPS VII exhibits a wide phenotypic spectrum, ranging from severe prenatal presentations with nonimmune hydrops fetalis (NIHF) and high early mortality to attenuated phenotypes with survival into adolescence or adulthood. In a cross‐sectional analysis of published cases, the median age of disease onset was the first day of life and the median age at diagnosis was 11 months. Clinical symptoms become more prominent at 3–4 years of age. Only a small number of patients with MPS VII have been reported to survive in adulthood, and these cases are generally associated with milder phenotypes. Nevertheless, the median survival for postnatally diagnosed patients remains approximately 30 years [4]. There are, however, also reports of individuals diagnosed in later adulthood, with stable disease [5], or individuals with extremely mild phenotypes [6].

Compared to other MPS subtypes, MPS VII is distinguished by its rarity and its relatively high association with NIHF, which is less frequently observed in other MPS disorders. Nonetheless, other clinical symptoms, such as skeletal dysplasia, organomegaly, and progressive multisystem involvement, resemble MPS I and MPS II [1].

Diagnosis of MPS VII typically follows a tiered approach. Clinical suspicion for MPS VII prompts further biochemical testing, including urinary GAG analysis and measurement of β‐glucuronidase activity. In patients with MPS VII, this typically reveals elevated DS and HS, with or without elevated CS, in combination with reduced beta‐glucuronidase activity. Molecular genetic testing is performed to confirm the presence of genetic variants in *GUSB*, the gene encoding β‐glucuronidase. As MPS VII is inherited in an autosomal recessive manner, disease manifestation requires pathogenic variants affecting both alleles of *GUSB* [2]. In siblings of individuals with known pathogenic variants, targeted genetic testing may facilitate a faster diagnosis, with subsequent metabolic evaluation as indicated.

Marked genotypic heterogeneity underlies the wide clinical variability observed in MPS VII. Locus‐specific variant databases have documented hundreds of unique variants in the *GUSB* gene, the majority of which are missense variants and many of which are currently classified as variants of uncertain significance (VUS) [7, 8, 9]. Despite the identification of pathogenic *GUSB* variants, genotype–phenotype correlations remain weak, with residual β‐glucuronidase activity correlating with disease severity and survival only in cohort‐based analyses [4, 9].

Beyond classical clinical and biochemical clues, peripheral blood smear morphology can provide an important, sometimes incidental diagnostic hint. The Alder–Reilly anomaly, marked by the presence of aberrant granulation in neutrophils (and variably in lymphocytes/monocytes), reflects intracellular GAG accumulation in leukocytes and is associated with several MPS subtypes [10, 11, 12, 13, 14, 15, 16, 17]. Although not disease‐specific, awareness of this finding helps timely metabolic evaluation for mucopolysaccharidoses, including MPS VII, and prevents misinterpretation as toxic granulation in inflammatory states.

Here, we report the case of a 31‐year‐old otherwise healthy woman who was incidentally diagnosed with MPS VII. She exhibited no typical clinical manifestations; however, the presence of the Alder–Reilly anomaly in the peripheral blood smear examination prompted further metabolic and genetic evaluation. The patient provided informed consent for the publication of this report.

## 2 Methods

### 2.1 Routine Laboratory Testing

All laboratory analyses were performed within an ISO 15189‐accredited laboratory following standard operating procedures (SOPs).

A complete blood count (CBC) was performed using a Sysmex XN‐series hematology analyzer (Sysmex, Kobe, Japan). The analyzer generated the flags: “abnormal WBC scattergram,” “abnormal lymphocytes,” and “giant platelets,” prompting preparation of a peripheral blood smear for further morphological assessment. Peripheral blood smears were prepared using the Sysmex SP‐10 slide maker/stainer, which performed automated slide preparation and May‐Grünwald staining according to validated laboratory settings. The stained slides were subsequently scanned and preclassified using the CellaVision DI‐60 module. Final morphological evaluation, including verification and correction of the automated preclassification, was performed by trained laboratory specialists.

Chemistry tests were performed on the Siemens Atellica Solution (Siemens Healthineers, Erlangen, Germany). C‐reactive protein (CRP), ferritin, vitamin B12, and folate were analyzed using manufacturer‐validated immunoassay methods according to SOPs.

### 2.2 Measurement of GAGs

Upon suspicion of a lysosomal storage disorder, urinary GAGs were measured. Samples were stored at −20°C until analysis, then thawed and subjected to GAG analysis. In this analysis, HS, DS, and keratan sulfate (KS) in urine were measured using a previously described method [18]. Briefly, samples were subjected to enzymatic digestion, and the resulting disaccharides were mixed with an internal standard (4UA‐2S‐GlcNCOEt‐6S, HD009, Iduron, UK). The disaccharides were quantified by UPLC‐MS/MS using an HS/DS/KS disaccharide calibration mix. Total HS was calculated as the sum of D0A0, D0S0, D0A6 + D2A0, and D0S6 + D2S0 disaccharides; total DS as the sum of D0a4 and D0a10; and total KS as the sum of g0A6 and g6A6. All measurements were corrected for urinary creatinine concentration. Analytical performance was monitored using quality control samples at two concentration levels.

### 2.3 β‐Glucuronidase Activity

Leukocytes were isolated from EDTA blood using standard procedures. The frozen cell pellet of leukocytes was suspended in 300 μL ice cold water and sonicated for 7 s under constant cooling in ice‐water. Protein concentration in the homogenate was determined by the copper‐reduction method using bicinchoninic acid, essentially as described by Smith et al. [19].

The activity of β‐glucuronidase was determined using the artificial substrate 4‐methylumbelliferyl (4MU)‐β‐D‐glucuronide essentially as described before [20, 21]. In short, the reaction mixture contained an aliquot of cell sample (10 μg), 0.1 M sodium acetate (pH 4.8), and 6 mM 4MU‐β‐D‐glucuronide in a total volume of 150 μL. After 30 min at 37°C, the reaction was terminated by the addition of 1350 μL 0.2 M sodium carbonate/glycine (pH 10.5), and the fluorescence of liberated 4MU was measured using a fluorometer (excitation 360 m, emission 450 nm). The concentration of 4MU was calculated using a calibration curve of 4MU standards.

### 2.4 Confirmatory Genetic Analysis

Molecular analysis of the *GUSB* gene was performed using 150 bp paired‐end Illumina sequencing and analyzed using the RoDa5 pipeline and Emedgene Analyze v37 (Illumina). Analysis covered coding sequences and splice regions.

### 2.5 Clinical Assessment

A structured medical history and physical examination were conducted following institutional internal medicine protocols. Particular attention was given to clinical features associated with mucopolysaccharidosis, including subtle dysmorphic facial characteristics, stature, skeletal deformities, joint mobility and contractures, hepatosplenomegaly, heart murmurs, and auditory or visual abnormalities.

### 2.6 Imaging Studies

Radiological studies, including skeletal imaging and abdominal ultrasonography, were performed in accordance with standard diagnostic procedures and reviewed by subspecialized radiologists to assess for findings characteristic of MPS (e.g., dysostosis multiplex or organomegaly). Echocardiography was performed by a trained technician under supervision of a licensed cardiologist.

## 3 Results

### 3.1 Presentation

A 31‐year‐old woman presented to her general practitioner with generalized fatigue. She and her partner had been trying to conceive for over a year without success.

### 3.2 Initial Laboratory Evaluation

A biochemical and hematologic work‐up to evaluate common causes of fatigue was conducted. CBC revealed mild anemia with hemoglobin of 7.2 mmol/L (reference range: 7.5–10.0 mmol/L). There were no signs of inflammation, nor nutritional insufficiencies (Table 1).

**TABLE 1:** Results of the initial hematologic and biochemical work‐up.

Laboratory test	Result	Reference interval	Unit
Hemoglobin	7.2	7.5–10.0	mmol/L
MCV	79	80.0–100.0	fL
MCH	1.490	1.700–2.100	fmol
MCHC	19.0	19.0–22.5	mmol/L
Erythrocytes	4.82	4.00–5.00	×10^12^/L
RDW‐SD	42.2	35–47	fL
Platelets	269	150–400	×10^9^/L
Leukocytes	5.1	4.0–10.0	×10^9^/L
CRP	< 0.5	0–5	mg/L
Ferritin	45	15–290	μg/L
Vitamin B12	263	150–675	pmol/L
Folate	29.1	10.0–45.0	nmol/L

Besides some nonalarming abnormalities in numerical parameters, the hematology analyzer generated several morphology‐related flags. Specifically, flags for “abnormal WBC scattergram,” “abnormal lymphocytes,” and “giant platelets” were triggered, prompting manual microscopic review.

### 3.3 Peripheral Blood Smear Findings

Microscopy demonstrated striking cytoplasmic inclusions: dense blue‐purple granules in all eosinophils, with similar (although less pronounced) granules in all other types of leukocytes, that is, neutrophils, lymphocytes, basophils, and monocytes (Figure 1A–D). In consultation with the patient's general practitioner, and given the lack of clinical manifestations, conservative management with deferred reassessment was agreed upon. Repeated hematologic evaluation, performed several months later, revealed an identical granule pattern. The characteristic distribution and appearance of the granules prompted suspicion of the Alder–Reilly anomaly, and the patient was referred to the department of adult metabolic diseases for evaluation.

**FIGURE 1:**
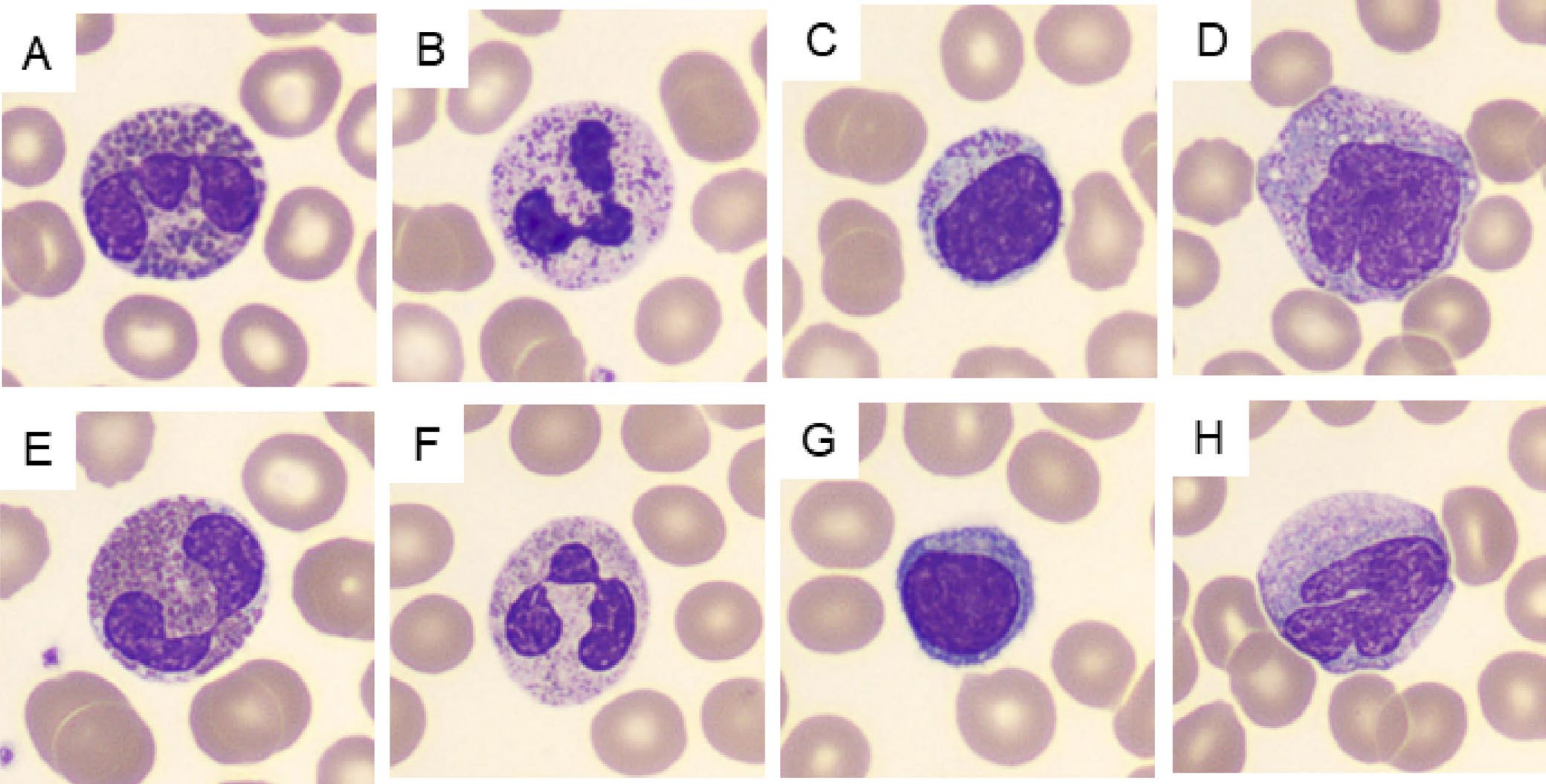
Alder–Reilly anomaly. Representative images of the Alder–Reilly anomaly in this patient (A–D) versus normal cells (E–H) in eosinophils (A, E), neutrophils (B, F), lymphocytes (C, G), and monocytes (D, H).

### 3.4 Urinary GAG Quantification and Leukocyte β‐Glucuronidase Activity

Urinary GAG analysis revealed a mild elevation of HS (1010 μg/L, 532 μg/mmol creatinine, reference range: 0–323 μg/mmol creatinine), corresponding to approximately 1.5× the upper limit of normal (ULN). DS was also moderately increased (230 μg/L, 121 μg/mmol creatinine, reference range: 0–53 μg/mmol creatinine), equivalent to 2.3× ULN. KS levels were within the normal range (143 μg/L, 75 μg/mmol creatinine, reference range: 0–110 μg/mmol creatinine).

Leukocyte β‐glucuronidase activity was markedly reduced, measured at 11 nmol/mg/h (reference range: 317–608 nmol/mg/h), corresponding to residual activity of 2%–3% of controls. The pronounced decrease in β‐glucuronidase activity, in combination with mild GAG elevation in urine, prompted mutation analysis of the *GUSB* gene.

### 3.5 GUSB Gene Analysis

Molecular analysis of the *GUSB* gene identified two heterozygous *GUSB* variants. The first variant, c.454G>A (p.Asp152Asn), results in a conservative amino acid substitution (Grantham score: 23 [0–215]). The variant was detected 2925 times (including 2 homozygotes) among 1 613 278 reference alleles in the Genome Aggregation Database (gnomADv4.1.0; http://gnomad.broadinstitute.org). Previous reports indicate that this variant causes reduced enzyme activity in the absence of clinical symptoms, consistent with a so‐called pseudodeficiency [22]. Therefore, this variant was classified as a class 3 variant of uncertain significance. The second variant, c.133_134del (p.Trp45Glufs*8), introduces a premature stop codon predicted to result in truncation of the β‐glucuronidase protein. This variant has not been previously reported and was not identified among > 1.6 million reference alleles in gnomAD (v4.1.0; http://gnomad.broadinstitute.org). Therefore, this variant was classified as a likely pathogenic class 4 variant. To assess whether the two variants were inherited in trans, both parents were sequenced. This analysis showed that each parent carried one of the genetic variants. Notably, neither parent exhibited the Alder–Reilly anomaly.

Together, these findings confirm compound heterozygosity *in trans* for two *GUSB* variants: a novel frameshift variant classified as likely pathogenic, and a class 3 variant that has previously been associated with pseudodeficiency (i.e., reduced enzyme activity in the absence of clinical disease).

### 3.6 Clinical Phenotyping

In‐depth clinical and radiologic phenotyping revealed no overt signs of MPS VII. The patient was a well‐functioning adult with normal neurological and motor development. She had congenital bilateral hearing loss due to a cochlear disorder, not otherwise specified, and used hearing aids. Results from serial audiological examinations indicated stable hearing over time. Notably, both her father and her uncle also had congenital hearing loss, not otherwise specified. This is suggestive of familial/genetic hearing loss, but not of autosomal recessive inheritance (there was no history of consanguinity). She had undergone tonsillectomy in childhood due to recurrent throat infections, but otherwise there was no history of recurrent infections or medical issues.

She had no current visual, respiratory, cardiac, abdominal, or skeletal complaints. Final height (165 cm) was close to the mid‐parental height prediction (168 cm). Physical and radiological examinations, including pelvis and spine radiographs, demonstrated normal joint mobility, no skeletal deformities/dysostosis multiplex, and no hepatosplenomegaly. Echocardiography showed normal cardiac anatomy and function, without valvular thickening and trace mitral valve regurgitation.

## 4 Discussion

MPS VII leads to the accumulation of partially degraded GAGs in lysosomes of many cell types, driving cellular dysfunction. MPS VII is ultrarare, with variable age of onset and widely variable symptomatology. Reported presentations range from severe neonatal presentations such as hydrops fetalis to milder, slowly progressive forms presenting in adulthood. Common clinical manifestations include short stature, skeletal dysplasia, hepatosplenomegaly, hernias, cardiac involvement, pulmonary insufficiency, and cognitive impairment. Here, we present a patient in whom abnormal leukocyte granulation was noted during routine laboratory testing. This patient did not exhibit any clinically apparent symptoms of MPS VII, but metabolic and genetic testing demonstrated elevated urinary GAGs, reduced β‐glucuronidase activity, and two heterozygous *GUSB* variants in trans (compound heterozygosity). Because MPS VII is ultrarare, published adult case reports remain limited, particularly for individuals identified incidentally in the absence of overt clinical manifestations.

A recent abstract, presented at the 2025 Annual Clinical Genetics Meeting (ACGM), identified 329 unique *GUSB* variants among 235 individuals with MPS VII carrying ≥ 1 *GUSB* variant, of which 67% were classified as VUS [7]. Among known pathogenic variants, substantial heterogeneity has been documented, potentially contributing to the wide phenotypic variability of MPS VII [9]. Severe phenotypes are most commonly associated with truncating mutations (e.g., frameshift, nonsense, splice‐site variants) or missense variants affecting conserved residues critical for protein stability or the active site [9].

Here, we identified two heterozygous *GUSB* variants in one patient: c.454G>A (p.Asp152Asn) and c.133_134del (p.Trp45Glufs*8), confirmed to be *in trans*. The missense variant p.Asp152Asn (p.D152N) has been previously associated with reduced enzymatic activity, but also with pseudodeficiency. It was originally described in the clinically unaffected, pseudodeficient mother of an MPS VII patient, occurring on the nonpathogenic allele, while the disease‐causing allele carried p.Leu176Phe [22]. β‐glucuronidase activity was reported to be approximately 6%–10% of control values. Screening of 100 healthy individuals identified one additional carrier. Importantly, functional expression studies demonstrated that cells transfected with cDNA encoding the p.Asp152Asn β‐glucuronidase variant undergo accelerated intracellular degradation, resulting in reduced enzymatic activity in vitro. Despite these measurable biochemical abnormalities, carriers remained clinically unaffected, leading to its classification as a pseudodeficiency allele [6, 22].

In contrast, the c.133_134del frameshift variant in this patient introduces a premature stop codon and is predicted to result in a truncated, nonfunctional enzyme or undergo nonsense‐mediated decay. Such truncating variants are well‐established null alleles in MPS VII. The combination of a null allele with a pseudodeficiency allele raises the question of how this genotype relates to the observed phenotype.

The markedly reduced leukocyte β‐glucuronidase activity (11 nmol/mg/h; reference 317–608 nmol/mg/h) that we observed in our patient corresponds to approximately 2%–3% of normal. Previous reports consistently demonstrate a strong relationship between residual β‐glucuronidase activity and clinical severity in MPS VII. Early reports show that the original patient described by Sly et al. showed < 2% of normal fibroblast GUS activity and a severe phenotype [23], whereas the two mildly affected brothers later reported by Gitzelmann et al. retained approximately 10% residual fibroblast activity [24]. These observations were later substantiated by larger functional datasets. In their comprehensive mutation update, Tomatsu et al. showed that 8 of 11 severe phenotype variants retained < 3% of normal cDNA‐derived activity, while 13 of 14 attenuated phenotype variants exceeded this 3% threshold [9]. More recently, Zielonka et al. identified clinically meaningful survival thresholds, showing that patients with > 1.4% residual enzyme activity in fibroblasts survived significantly longer than patients with lower residual activity [4], further reinforcing the prognostic value of enzymatic activity. It must be noted that even though both fibroblasts and leukocytes can be used to measure residual activity in MPS, the absolute activity may differ and results are not directly interchangeable between these cell types due to differences in cellular composition, enzyme expression, and preanalytical variability; therefore, interpretation should always rely on matrix‐specific reference ranges [25].

Of particular relevance, Vervoort et al. specifically characterized the biochemical and functional consequences of the p.Asp152Asn pseudodeficiency variant. Their pseudodeficient individual exhibited ~5% leukocyte activity (and 10%–14% in fibroblasts) and remained clinically unaffected [6]. Together, the 2%–3% residual activity that we observed in leukocytes aligns with the presence of one pseudodeficiency‐like missense allele and one null allele, producing a combined residual activity that can protect against a severe phenotype [9].

We observed visible GAG storage (Alder–Reilly granules) across all leukocyte lineages. As lysosomes are a fundamental component of all nucleated cells, including all leukocyte subsets, accumulation of undegraded macromolecules may be detected in circulating blood cells in various lysosomal storage disorders [26]. Although the Alder–Reilly anomaly is a well‐recognized feature in mucopolysaccharidoses, including MPS VII, its relationship with disease severity remains uncertain, with no consistent correlation reported. Our findings illustrate that characteristic leukocyte changes can be present even in the absence of overt clinical manifestations.

The coexistence of markedly reduced enzyme activity, elevated urinary GAGs, and demonstrable lysosomal storage within leukocytes, in the absence of clinically apparent disease, suggests that residual β‐glucuronidase activity alone may not fully explain the observed phenotype. Additional genetic, epigenetic, or regulatory factors may influence disease expression in MPS VII. The mechanisms underlying this phenotypic variability remain to be elucidated.

The present case also raises questions regarding the role of enzyme replacement therapy (ERT) in individuals with marked biochemical abnormalities but no overt clinical symptoms. While vestronidase alfa has demonstrated clinical benefit in symptomatic patients with MPS VII, its potential benefit in asymptomatic individuals remains unknown. Given the absence of clinically relevant organ involvement despite extensive phenotypic evaluation, there is currently no evidence that treatment would provide a measurable clinical benefit in our patient. Therefore, careful longitudinal follow‐up may presently be more informative than initiating treatment based solely on biochemical abnormalities, particularly given the substantial costs and lifelong treatment burden associated with ERT.

All in all, this case illustrates an incidental diagnosis of MPS VII in an otherwise asymptomatic adult, in whom the presence of the Alder–Reilly anomaly in the peripheral blood leukocytes served as a key diagnostic clue. In our patient, a genotype‐derived residual β‐glucuronidase activity of approximately 2%–3% was sufficient to prevent overt organ involvement, yet inadequate to fully avert lysosomal storage within leukocytes, underscoring the value of peripheral blood morphology as an accessible indicator of impaired lysosomal GAG degradation.

The present case extends previous observations regarding the p.Asp152Asn pseudodeficiency allele. While this variant has previously been associated with reduced β‐glucuronidase activity in clinically unaffected individuals, we demonstrate that, when occurring in trans with a presumed null allele, it is associated not only with markedly reduced enzyme activity but also with detectable lysosomal GAG accumulation in leukocytes. Despite these biochemical and morphological abnormalities, no clinical manifestations of MPS VII have been observed to date. Whether this genotype represents true pseudodeficiency or an extremely attenuated form of MPS VII remains uncertain. Long‐term follow‐up will be required to determine whether this degree of residual activity is sufficient to prevent disease manifestations throughout life.

## Author Contributions

Conception and design: Margaretha G.M. Roemer, Kasper W. ter Horst, and Janneke C. Zant. Analysis and interpretation of data: Margaretha G.M. Roemer, Monique Chevallier, Marie van Dijk, André B.P. van Kuilenburg, Kasper W. ter Horst, and Janneke C. Zant. Drafting the manuscript: Margaretha G.M. Roemer. Critically revising the manuscript: Monique Chevallier, Marie van Dijk, André B.P. van Kuilenburg, Kasper W. ter Horst, and Janneke C. Zant. Supervising the study: Janneke C. Zant.

## Funding

The authors have nothing to report.

## Ethics Statement

All patient information was de‐identified according to institutional privacy guidelines. In accordance with local research policies, formal ethics committee approval was not required for publication of a single anonymized clinical case. Written informed consent for publication was obtained from the patient.

## Conflicts of Interest

The authors declare no conflicts of interest.

## Data Availability

The data that support the findings of this study are available from the corresponding author upon reasonable request.
